# Why Is a True Corpus Luteum Formed Only in Cases of Impregnation

**Published:** 1852-03

**Authors:** Alexander Harvey


### ANATOMY AND PHYSIOLOGY.

*Why is a true Corpus Luteum formed only in cases of Impregnation.* By Dr. Alexander Harvey.—Taking it for granted that the assumption is a true one that a *genuine* corpus luteum does only occur in impregnation, though a *false* one appears after each oviposit, the author seeks for a reason, and finds one in the following explanation :

1. When a mature ovum is impregnated, provision is forthwith made to retain it in utero, and both ovum and uterus become the seat of active vital processes, which cause a prolonged determination of blood to the reproductive organs. The ruptured ovisac participates in this afflux and the organizable deposit which it contains is placed under circumstances favorable to the exercise of its inherent powers of growth, and thus it becomes developed into a true corpus luteum.

2. On the other hand, when the ovum is not impregnated, it is thrown off in the menstrual discharge, no prolonged afflux taking place as in the former case; the organizable deposit is not favorably placed for further development, and a *false* corpus luteum is the consequence.—*Monthly Jour. of Med. Scien. from Prov. Med. and Surg. Jour.*

